# Axis-Specific Peripartum Management for Radiation-Induced Panhypopituitarism With Arginine Vasopressin Deficiency: A Case Report

**DOI:** 10.1016/j.aed.2026.01.006

**Published:** 2026-01-19

**Authors:** Takashi Kono, Hiroka Miyagawa, Yuto Kawauchi, Yuki Taki, Ikki Sakuma, Naoko Hashimoto, Yutaka Oki, Akira Shimatsu, Tomoaki Tanaka

**Affiliations:** 1 Department of Molecular Diagnosis, Chiba University Graduate School of Medicine, Chiba, Japan; 2 Research Institute of Disaster Medicine, Chiba University, Chiba, Japan; 3 Second Department of Internal Medicine, Hamamatsu University School of Medicine, Shizuoka, Japan; 4 Diabetes and Endocrinology Center, and Academic Advisor, Hamamatsu-kita Hospital, Hamamatsu, Japan; 5 Advanced Medical Care Center, Omi Medical Center, Shiga, Japan

**Keywords:** central diabetes insipidus, FT4-guided levothyroxine, intrapartum stress-dose, panhypopituitarism, pregnancy

## Abstract

**Background/Objective:**

We report the perinatal course and practical, axis-specific management of a 34-year-old woman with panhypopituitarism and arginine vasopressin deficiency (AVP-D) consequent to cranial irradiation and ifosfamide, cisplatin, and etoposide chemotherapy for a germinoma who conceived via in vitro fertilization.

**Case Report:**

Care was organized by endocrine axis with coordinated obstetric collaboration. Subcutaneous growth hormone was discontinued upon pregnancy confirmation. Central hypothyroidism was managed using free thyroxine targets with trimester-appropriate oral levothyroxine dose adjustments. Secondary adrenal insufficiency was addressed with oral hydrocortisone and a predefined intrapartum stress-dose intravenous hydrocortisone plan. AVP-D was managed by continuing oral desmopressin with symptom-guided monitoring. Labor was electively induced at term, and the peripartum course was uncomplicated. Maternal and umbilical cord endocrine profiles at delivery were documented to contextualize axis physiology. The newborn had reassuring adaptation and normal early pediatric assessments.

**Discussion:**

This case illustrates that a pragmatic, axis-wise strategy centered on free thyroxine-guided thyroid replacement, explicit glucocorticoid stress coverage, and disciplined AVP-D monitoring can be safely implemented in collaboration with reproductive medicine and obstetrics after cranial irradiation and ifosfamide, cisplatin, and etoposide chemotherapy.

**Conclusion:**

A reproducible, axis-specific pathway may support safe pregnancy and delivery in women with complex pituitary sequelae, provided that monitoring plans and intrapartum stress-dose coverage are defined in advance.

**Highlights**

• Central hypothyroidism: titrate levothyroxine to the upper-normal free thyroxine range because thyroid-stimulating hormone is unreliable; make small trimester-appropriate adjustments, and reassess promptly postpartum
• Adrenal insufficiency: use a written intrapartum stress-dose protocol (eg, hydrocortisone 100 mg IV at induction, then 50 mg q8h) with a 24–48-hour taper to maintenance; avoid chronic over-replacement
• Arginine vasopressin deficiency: continue desmopressin with symptom-guided timing; monitor serum sodium and osmolality; routine obstetric fluids and oxytocin are feasible under coordinated monitoring
• Care model: plan early cross-disciplinary management (endocrinology–reproductive medicine–obstetrics), schedule term induction when appropriate, and document maternal and cord-blood hormones to contextualize physiology

**Clinical Relevance**

This case reports how a pragmatic, axis-specific pathway—free thyroxine-guided levothyroxine, predefined intrapartum hydrocortisone with brief postpartum taper, and symptom-guided desmopressin with electrolyte monitoring—enabled an uncomplicated term delivery in radiation-induced panhypopituitarism with arginine vasopressin deficiency.

## Introduction

Pregnancy in women with radiation-induced panhypopituitarism, particularly when complicated by arginine vasopressin deficiency (AVP-D), is rare and clinically challenging.^1^ Management requires adapting axis-specific targets to gestational physiology: titrating levothyroxine to the upper-normal free thyroxine (FT4) range (thyroid stimulating hormone [TSH] is unreliable),^2^, ^3^, ^4^ ensuring an anticipatory intrapartum stress-dose plan for adrenal insufficiency,^5^^,^^6^ and continuing symptom-guided desmopressin for AVP-D.^7^^,^^8^ Despite these principles, integrated peripartum evidence remains limited.^9^ We report the successful management of an in vitro fertilization (IVF) pregnancy in a woman with radiation-induced PH and AVP-D using a pragmatic, axis-wise pathway, including maternal and cord-blood hormone profiles.

## Case Report

**Fig. 1:**
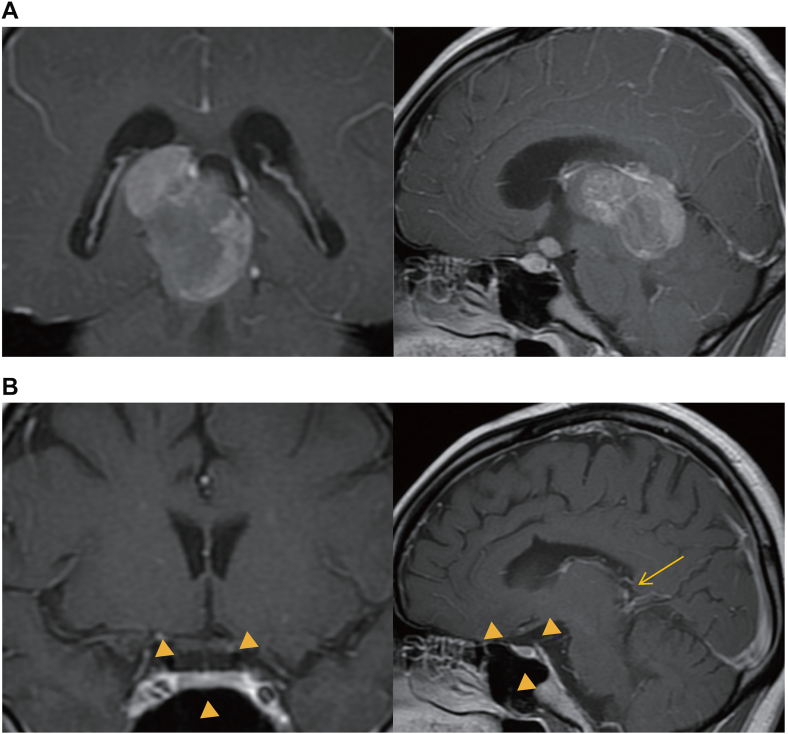
MRI findings at initial diagnosis and before conception. (A) MRI at initial diagnosis showing the germinoma in the pineal and suprasellar regions. (B) Preconception MRI showing resolution of the tumor after treatment. Arrowheads indicate the pituitary stalk and sella, while the arrow indicates the treated pineal lesion; the optic chiasm shows no compressive changes. *MRI* = magnetic resonance imaging.

A 34-year-old woman (gravida 0, para 0) with panhypopituitarism and AVP-D, which developed as a consequence of cranial irradiation (total dose of 46 Gy) and ICE chemotherapy (ifosfamide, cisplatin, and etoposide) for a germinoma at age 20. The ICE regimen consisted of 3 courses of ifosfamide (900 mg/m^2^), cisplatin (20 mg/m^2^), and etoposide (60 mg/m^2^) administered intravenously for 5 consecutive days per course, sought pregnancy via IVF. The endocrine profile of the patient at the time of initial diagnosis (Table 1) confirmed panhypopituitarism and also showed mild hyperprolactinemia (prolactin, 54.4 ng/mL), likely consistent with a stalk effect in the context of prior cranial irradiation and treated sellar disease. The patient had been on a stable replacement regimen (hydrocortisone, 17.5 mg; levothyroxine, 100 μg; desmopressin, 60 μg; and growth hormone, 0.5 mg) for several years. Notably, the patient had secondary amenorrhea, requiring cyclic estrogen-progestin therapy to induce withdrawal bleeding. The patient reported no recent headache or visual symptoms. Baseline magnetic resonance imaging before conception showed resolution of the prior sellar lesion (consistent with remission) after treatment (Fig. 1). A multidisciplinary team (endocrinology, reproductive medicine, and obstetrics) planned axis-specific adjustments for conception, pregnancy, and delivery.

**Table 1:** Endocrine Profile at the Time of Initial Diagnosis of Panhypopituitarism

Parameter	Baseline [SI]	Normal range [SI]
TSH	1.73 mIU/L	0.5–5.0 mIU/L
FT3	2.00 pg/mL [3.08 pmol/L]	2.3–4.0 pg/mL [3.54–6.16 pmol/L]
FT4	0.52 ng/dL [6.69 pmol/L]	0.9–1.7 ng/dL [11.6–21.9 pmol/L]
Basal GH	0.36 ng/mL	Stimulation test peak >3.0 ng/mL
IGF-1/SD	75.3 ng/mL/−4.2	182–780 ng/mL
LH	0.3 mIU/mL	2.4–12.6 mIU/mL
FSH	1.1 mIU/mL	1.4–9.9 mIU/mL (follicular)
E2	11.2 pg/mL [41.1 pmol/L]	30–400 pg/mL [110.0–1468.0 pmol/L] (premenopausal)
PRL	54.4 ng/mL [1153.0 mIU/L]	3–27 ng/mL [63.6–572.0 mIU/L] (females)
ACTH	18.6 pg/mL [4.09 pmol/L]	10–60 pg/mL [2.2–13.2 pmol/L]
Cortisol	4.9 μg/dL [135.0 nmol/L]	8–25 μg/dL (morning)
AVP	0.4 pg/mL [0.37 pmol/L]	0.9–4.6

Abbreviations: AVP = arginine vasopressin; ACTH = adrenocorticotropic hormone; E2 = estradiol; FSH = follicle-stimulating hormone; FT3 = free triiodothyronine; FT4 = free thyroxine; GH = growth hormone; IGF-1 = insulin-like growth factor-1; LH = luteinizing hormone; PRL = prolactin; SD = standard deviation; TSH = thyroid-stimulating hormone.

Values in Table 1 represent basal hormone levels at the initial diagnosis (age 18). At this stage, a combined anterior pituitary stimulation test (GRH, CRH, and TRH) showed a preserved GH response (Peak 15.61 ng/mL), indicating preserved pituitary reserve. However, IGF-1 was markedly suppressed (SDS -4.2). This discrepancy suggested hypothalamic GH deficiency (impaired endogenous GHRH supply) secondary to the pituitary stalk lesion. Severe GH deficiency was subsequently confirmed clinically by the persistent suppression of IGF-1 following multimodal treatment for germinoma.

Values in conventional units; SI values in [].

Preconception and fertility treatment: Before IVF, thyroid replacement was titrated to target FT4 within the upper-normal range (target approx. 1.2–1.4 ng/dL), acknowledging the unreliability of TSH in central hypothyroidism.^2^, ^3^, ^4^ Adrenal replacement was confirmed adequate by clinical assessment and morning symptoms, with written instructions for stress dosing. AVP-D was controlled with desmopressin; the patient was taught symptom-guided timing and to avoid overcorrection. Controlled ovarian stimulation was performed with human menopausal gonadotropin (hMG, 150 IU) and human chorionic gonadotropin trigger, followed by luteal support with transdermal estradiol and vaginal progesterone until 8 weeks and 5 d of gestation. Regarding ovarian function, although the ICE chemotherapy and prior history of ovarian cyst enucleation at age 19 posed a risk for primary ovarian insufficiency, the patient successfully produced 6 oocytes (5 of which were fertilized) during her third retrieval cycle at age 33. This response suggests that despite potential chemotherapy-induced gonadotoxicity, sufficient ovarian reserve was preserved to allow for successful assisted reproduction. After a total of 3 oocyte retrieval cycles and 5 frozen embryo transfer attempts, clinical pregnancy was achieved uneventfully.

### Early Pregnancy (≤12 weeks)

**Fig. 2:**
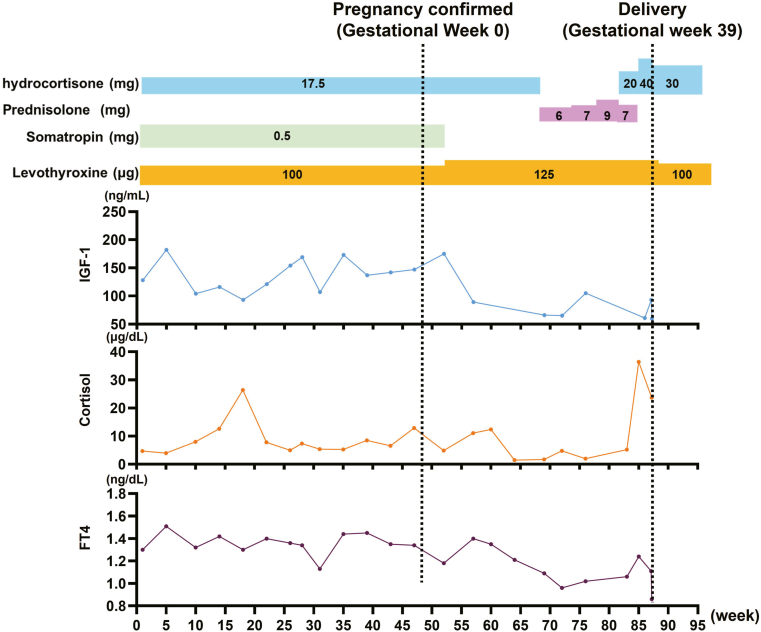
Longitudinal perinatal profiles spanning fertility treatment, gestation, and delivery. Longitudinal perinatal profiles spanning fertility treatment, gestation, and delivery. The X-axis represents weeks from the start of fertility treatment and monitoring. Vertical markers denote conception (gestational week 0) and delivery (gestational week 39). Maternal IGF-1 dynamics are physiologically driven in part by placental growth hormone during pregnancy. Serum cortisol levels are presented for longitudinal clinical reference but were not utilized as targets for dose titration, as these measurements are confounded by oral hydrocortisone replacement and pregnancy-induced elevations in cortisol-binding globulin. *IGF-1* = insulin-like growth factor 1; *FT4* = free thyroxine.

At 6 gestational weeks, the care plan emphasized 1) maintaining FT4 in the upper-normal range via levothyroxine adjustment^2^^,^^3^, 2) confirming euadrenal status while avoiding excessive glucocorticoid administration, and 3) continuing desmopressin with vigilance for hyponatremia. The patient reported stable energy and thirst/urine patterns. Electrolyte levels were within range at routine checks.^7^^,^^8^ Nausea and vomiting were mild. The patient was continued on the baseline maintenance hydrocortisone dose (17.5 mg/d) but was instructed to take an additional 5–10 mg as a rescue dose in the event of exacerbated fatigue or nausea (sick-day management). Longitudinal perinatal hormone and replacement profiles spanning fertility treatment, gestation, and delivery are shown in Figure 2.

### Mid-to-late Pregnancy (13–36 weeks)

During the second and third trimesters, thyroid replacement remained guided by FT4^4^; values were kept in the upper-normal range with small stepwise dose adjustments as gestation advanced. Maternal insulin-like growth factor 1 (IGF-1) level typically rises across mid-to-late gestation under the influence of placental growth hormone, providing a physiologic context for the observed trajectory.^10^, ^11^, ^12^ Clinical euadrenal status was preserved without intercurrent illness. Blood pressure and weight gain followed expected trajectories (eg, blood pressure remained stable approx. 110–120/70–80 mmHg, with no peripheral edema); no features of preeclampsia or gestational diabetes were observed. AVP-D control remained stable on desmopressin, with occasional minor timing adjustments to match diurnal symptoms; serum sodium and osmolality remained within reference ranges. Fetal growth and anatomy scans were appropriate for gestational age, and nonstress testing later in pregnancy was reassuring.

### Peripartum (≥37 weeks)

With induction planned at 39 weeks, the oral glucocorticoid maintenance regimen was switched from prednisolone (7 mg daily) to hydrocortisone in anticipation of delivery. This involved a brief overlap period where prednisolone 7 mg daily was co-administered with oral hydrocortisone 20 mg daily, after which prednisolone was discontinued, establishing a new oral maintenance dose of hydrocortisone 40 mg daily prior to labor. A stress-dose protocol was finalized in collaboration with obstetrics to mitigate the risk of adrenal crisis.^5^^,^^6^ On the day of induction, stress-dose hydrocortisone (100 mg intravenously at labor induction, followed by 50 mg intravenously every 8 h during labor) was administered per protocol. Levothyroxine was maintained at the established pregnancy dose. Desmopressin was continued with careful fluid and electrolyte monitoring. Labor became hypotonic and was successfully augmented with synthetic oxytocin, with continuous fetal monitoring. Delivery was uncomplicated; the neonate had appropriate birth weight and Apgar scores. No peripartum electrolyte disturbances or hemodynamic instability were observed. Maternal and cord-blood endocrine values at delivery are summarized in Table 2. Where relevant, cord-blood endocrine values were interpreted against established neonatal reference intervals (Japanese IGF-1 and biochemical data), with classic cord neuroendocrine measurements used for historical context.^13^, ^14^, ^15^

**Table 2:** Maternal vs Cord Blood Endocrine Profile at Delivery

Hormone	Maternal blood (value)	Cord blood (value)	Maternal blood (3rd trimester)	Cord blood (reference)
GH	11.5 ng/mL [11.5 μg/L]	69.6 ng/mL [69.6 μg/L]	1–14 ng/mL [1–14 μg/L]	See footnote
IGF-1	93 ng/mL [12.16 nmol/L]	50 ng/mL [6.54 nmol/L]	109–265 ng/mL [14.25–34.65 nmol/L]	9–71 ng/mL [1.18–9.28 nmol/L] (10th–90th percentile)
PRL	37.5 ng/mL [795 mIU/L]	291 ng/mL [6169 mIU/L]	100–350 ng/mL [2120–7420 mIU/L]	See footnote
TSH	0.006 mIU/L	11.3 mIU/L	0.2–4 mIU/L	See footnote
FT3	2.04 pg/mL [3.14 pmol/L]	1.41 pg/mL [2.17 pmol/L]	1.8–4.2 pg/mL [2.76–6.45 pmol/L]	See footnote
FT4	1.11 ng/dL [14.29 pmol/L]	1.23 ng/dL [15.83 pmol/L]	0.8–1.9 ng/dL [10.3–24.45 pmol/L]	See footnote
ACTH	2.9 pg/mL [0.6 pmol/L]	110 pg/mL [24.2 pmol/L]	10–60 pg/mL [2.2–13.2 pmol/L]	See footnote
Cortisol	55.6 μg/dL [1534 nmol/L]	105 μg/dL [2897 nmol/L]	15–90 μg/dL [414–2483 nmol/L]	See footnote

Abbreviations: ACTH = adrenocorticotropic hormone; FT3 = free triiodothyronine; FT4 = free thyroxine; GH = growth hormone; IGF-1 = insulin-like growth factor-1; PRL = prolactin; TSH = thyroid-stimulating hormone.

Reference values and sources.

- Maternal reference ranges: Age-appropriate normal values for the third trimester.

- Neonatal reference ranges: Japanese cord blood reference data (eg, refs 13–15).

- GH/PRL: Neonatal hypersecretion on the day of birth with wide inter-individual range.

- IGF-1: Japanese umbilical cord percentiles (10th–90th: 9–71 ng/mL).

- TSH, FT3, FT4, cortisol: Japanese neonatal reference data.

- ACTH: Cord blood reference data.

### Postpartum Course and Follow-up

After delivery, the intravenous hydrocortisone stress-dose was tapered off over the first 24–48 h. The patient was transitioned to a new oral postpartum maintenance dose of hydrocortisone 30 mg daily. Education on sick-day rules was reinforced. Levothyroxine was reassessed postpartum with FT4-guided adjustments toward the nonpregnant target range.^2^ Desmopressin dosing was returned to the prepregnancy schedule; serum sodium remained normal. The patient reported satisfactory recovery. Postpartum lactation was minimal and insufficient for nursing, consistent with the low prolactin levels. Early pediatric assessments of the infant showed normal growth and development.

## Discussion

Under coordinated endocrine–obstetric care, an axis-specific pathway supported an uncomplicated peripartum course and favorable neonatal outcomes in a woman with radiation-induced panhypopituitarism and AVP-D following an IVF pregnancy. This pathway comprised 3 components: FT4-guided levothyroxine, a predefined intrapartum hydrocortisone stress-dose protocol, and continued desmopressin with symptom-guided monitoring. Maternal and cord blood endocrine profiles at delivery are provided to contextualize axis physiology.

Thyroid management is critical, as TSH is unreliable in central hypothyroidism; levothyroxine must be titrated to maintain FT4 in the upper-normal range (eg, 1.2–1.4 ng/dL).^2^, ^3^, ^4^ In our patient, whose FT4 at diagnosis was 0.52 ng/dL (Table 1), replacement was optimized to ∼1.4 ng/dL (Fig. 2) before IVF. Levels were maintained via stepwise adjustments, reaching 1.11 ng/dL at delivery (Table 2). This proactive approach obviated large late-trimester escalations. Postpartum reassessment is crucial as placental withdrawal rapidly alters requirements.^2^

Adrenal replacement during labor is critical to prevent adrenal crisis.^5^^,^^6^ Our predefined stress-dose protocol (hydrocortisone, 100 mg IV bolus; 50 mg q8h during labor) reflects a conservative approach given the unpredictability of labor augmentation.^5^ This protocol, followed by a prompt taper to maintenance (oral hydrocortisone, 30 mg), was associated with hemodynamic stability and an uncomplicated postpartum course. Equally important is avoiding chronic overreplacement; between acute stress events, clinical assessment and adverse-effect surveillance remain the primary tools for dose calibration.

AVP-D management remained straightforward by continuing desmopressin with careful monitoring.^7^^,^^8^ Dose stability was largely maintained as desmopressin is resistant to placental vasopressinase, which degrades endogenous vasopressin. Hyponatremia risk was mitigated by patient education on thirst-driven drinking rather than rigid fluid goals. The patient maintained stable electrolytes, suggesting routine obstetric fluids and oxytocin can be used safely with coordinated monitoring.

This case highlights the value of early crossdisciplinary planning, where IVF teams initiate endocrine targets and obstetrics aligns intrapartum execution, reducing uncertainty for complex cases.

Our patient had documented GH deficiency (IGF-1 75.3 ng/mL; Table 1). GH replacement was discontinued at 6 weeks gestation per local guidelines in Japan (GH therapy is not insured and contraindicated/cautioned in pregnancy), limiting its use despite some favorable European observational data.^10^, ^11^, ^12^^,^^16^^,^^17^ These observational studies have suggested that continuing replacement until placental growth hormone level rises, aiming to maintain IGF-1 in the normal range during the critical first trimester, may not increase maternal or fetal risk.^10^, ^11^, ^12^ In our patient, maternal IGF-1 levels remained low, rising modestly mid-gestation (reaching 93 ng/mL at delivery; Table 2), likely reflecting partial compensation by placental GH.^10^, ^11^, ^12^ However, IGF-1 levels remained below the reference range (109–265 ng/mL). Despite this, pregnancy and neonatal outcomes were favorable, demonstrating success is achievable without GH replacement if other axes are managed. Whether early GH continuation to normalize IGF-1 levels would optimize outcomes remains an open question.^9^

Additionally, the maternal prolactin level at delivery (37.5 ng/mL; Table 2) was relatively low compared with typical physiological ranges for the third trimester, possibly reflecting insufficient lactotroph reserve secondary to panhypopituitarism.

## Limitations

Limitations include the generalizability of a single case and the discontinuation of GH replacement in early pregnancy per local guidelines in Japan, which prevented assessment of its potential benefits on IGF-1 levels or fetal growth. We also did not measure placental GH-V levels or long-term offspring outcomes.

## Conclusion

An axis-specific, coordinated plan centered on FT4-guided levothyroxine, predefined intrapartum hydrocortisone coverage with brief postpartum taper, and symptom-guided desmopressin may support safe perinatal outcomes in pregnancies with radiation-induced panhypopituitarism and AVP-D.

## Data Availability

All data relevant to the case are included in the article and its tables/figures.

## Ethics Statement

Ethical review and approval were waived for this single-patient case report according to the policy of Chiba University, as this work does not meet the definition of human subjects research. All procedures complied with the provisions of the Declaration of Helsinki.

## Patient Consent

Written informed consent for publication of the case details and accompanying images was obtained from the patient.
